# Duck hepatitis A virus type 1 mediates cell cycle arrest in the S phase

**DOI:** 10.1186/s12985-022-01839-6

**Published:** 2022-06-27

**Authors:** Yuanzhi Liu, Yanglin Li, Mingshu Wang, Anchun Cheng, Xumin Ou, Sai Mao, Di Sun, Ying Wu, Qiao Yang, Renyong Jia, Bin Tian, Shaqiu Zhang, Dekang Zhu, Shun Chen, Mafeng Liu, Xinxin Zhao, Juan Huang, Qun Gao, Yanling Yu, Ling Zhang

**Affiliations:** 1 Institute of Preventive Veterinary Medicine, Sichuan Agricultural University, Wenjiang, Chengdu City, 611130 Sichuan China; 2 Key Laboratory of Animal Disease and Human Health of Sichuan Province, Sichuan Agricultural University, Wenjiang, Chengdu City, 611130 Sichuan China; 3 Avian Disease Research Center, College of Veterinary Medicine, Sichuan Agricultural University, Wenjiang, Chengdu City, 611130 Sichuan China

**Keywords:** Duck hepatitis A virus type 1, Cell cycle, S phase, Non-structural protein 3D

## Abstract

**Background:**

Duck hepatitis A virus type 1 (DHAV-1) is one of the most serious pathogens endangering the duck industry. However, there are few studies on the regulation of the cell cycle by DHAV-1.

**Methods:**

In this study, flow cytometry was applied to analyze the effect of DHAV-1 infection on the cell cycle of duck embryo fibroblasts (DEFs). Subsequently, we analyzed the effects of cell cycle phases on DHAV-1 replication by real-time reverse transcriptase quantitative PCR (real-time RT-qPCR).

**Results:**

Flow cytometry data analysis found that DEFs in the S phase increased by 25.85% and 54.21% at 24 h and 48 h after DHAV-1 infection, respectively. The levels of viral RNA detected by real-time RT-qPCR were higher in the DEFs with synchronization in the S phase or G0/G1 phase than in the control group. However, there was no difference in viral copy number between the G2/M phase arrest and control groups. In addition, non-structural protein 3D of DHAV-1 significantly increased cells in the S phase, indicating that 3D protein is one of the reasons for the cell cycle arrest in the S phase.

**Conclusions:**

In summary, DHAV-1 infection induces the cell cycle arrest of DEFs in the S phase. Both S phase and G0/G1 phase synchronization facilitate the replication of DHAV-1, and 3D protein is one of the reasons for the S phase arrest.

## Introduction

The cell replication cycle is divided into the G0 phase at rest, the intermitotic phase (G1 phase, S phase, and G2 phase), and the mitosis phase (M). The sequential replacement of each phase requires the participation of cyclin and cyclin-dependent kinase (CDK) in the cell. Cyclin E1 and CDK2 form a complex to regulate cell cycle transition from G1 to S phase [1]. CDK2 and Cyclin A regulate the progression from S to G2 phase [2]. CDK1 and Cyclin B are the main regulatory proteins in the M phase [3]. Regulating the host cell cycle is a common strategy exploited by the virus. In DNA viruses, there may be multiple cell cycle changes. Bocavirus minute virus of canines (MVC) arrests the S phase of cells in the early infection and arrests the G2/M phase of the cells in the late infection [4, 5]. Human parvovirus B19 (B19V) can block the cell cycle in the G2/M phase [6, 7]. However, Luo et al*.* found that B19V blocks the cell cycle in the S phase through infectious cloning and the 5-Bromo-2’-deoxyuridine (BrdU) method [8]. In addition, the nonstructural protein NS1 of B19V can also block the G1 and G2/M phases of cells [9, 10], suggesting that the process of DNA virus regulating the cell cycle is more complicated. In RNA viruses, there have been extensive reports on the research of Zika virus (ZIKV), Coxsackievirus A6 (CVA6), Coxsackievirus A16 (CVA16), and Enterovirus 71 (EV71) on cell cycle regulation [11–13]. Although different viruses block the cell cycle at different phases, the purpose of the virus to block the cell cycle is to create a favorable environment for its replication.

Duck hepatitis A virus type 1 (DHAV-1) belongs to the *Avihepatovirus* genus of the *Picornaviridae* family and is one of the most serious pathogens that harm young ducklings. After ducklings are infected with DHAV-1, the main pathological changes are in the liver, and the extremely scattered infection also occurs in the kidneys [14, 15]. Like other positive-sense single-stranded RNA viruses, its genome consists of a 5′ untranslated region (5′ UTR), an open reading frame (ORF), and a 3′ untranslated region (3′ UTR) [16]. The ORF is first translated into precursor polyprotein, which will be cleaved into structural protein and non-structural protein by viral protease 3C or 3CD. These viral proteins play an important role in viral life activities [17–23]. After the virus infects cells, cell apoptosis is often accompanied by alternating cell cycle progression [4, 24, 25]. DHAV-1 can induce apoptosis in cells and tissues [14, 19, 26]. However, the regulation of DHAV-1 on the cell cycle has not been reported yet.

In this study, we explored the effect of DHAV-1 infection on the DEFs cell cycle. Our results showed that DHAV-1 infection caused the DEFs cell cycle to be arrested in the S phase and synchronization in the S phase was beneficial to the replication of DHAV-1. Interestingly, G0/G1 phase arrest is also beneficial to DHAV-1 replication. In addition, we also proved that the non-structural protein 3D of DHAV-1 can cause cell cycle arrest in the S phase.

## Materials and methods

### Cells and viruses

The DHAV-1 H strain (GenBank: JQ301467.1) was provided by the Institute of Preventive Veterinary Medicine at Sichuan Agricultural University. The primary duck embryo fibroblasts (DEFs) were described previously [27]. DEFs were grown in a minimum essential medium (MEM) containing 10% newborn calf serum (Gibco) and incubated at 37 °C with 5% CO_2_ in an incubator. Then, DEFs were infected with DHAV-1 for 2 h, and the unbound virus was removed by washing with phosphate-buffered saline (PBS) twice before the cells were overlaid with MEM containing 2% newborn calf serum. UV-DHAV-1 is obtained by irradiating DHAV-1 with UV light with a wavelength of 253.7 nm for 6 h.

### Expression plasmids, antibodies, and reagents

The plasmid pCAGGS-3D-HA was constructed in a previous study [28]. Mouse anti-HA was purchased from MBL, mouse anti-β-actin antibody was purchased from TransGen Biotech, rabbit anti-VP3 antibody was prepared in our laboratory [29], and HRP-conjugated goat anti-mouse IgG and HRP-conjugated goat anti-rabbit IgG were purchased from Beyotime. Thymidine, Nocodazole, and Dimethyl sulfoxide were purchased from Sigma, and transfection reagent was obtained from TransGen Biotech.

### Cell cycle analysis by flow cytometry

After virus infection, drug treatment, or transfection, DEFs were digested with 0.25% trypsin (Gibco) and resuspended with pre-cooled PBS. Then, DEFs were centrifuged and added 75% cold ethanol to fix overnight. Before adding 500 μl PI/RNase Staining Buffer (BD Biosciences) for 15 min, DEFs were washed with pre-cooled PBS to remove ethanol. Finally, DEFs were filtered into a new centrifuge tube and analyzed by flow cytometry.

### Viral RNA load in DEFs

Total RNA was isolated using RNAiso Plus Reagent (TaKaRa) according to the manufacturer's instructions. The number of viral copies in total RNA was measured using methods previously established in our laboratory [30].

### Cell cycle synchronization

DEFs were treated with 1.0 mM Thymidine, serum-free medium, or 25 ng/ml Nocodazole for 24 h, and the cell cycle distribution was detected by flow cytometry.

### Western blot analysis

DEFs were transfected with pCAGGS-3D-HA expressing the 3D protein. Cells were lysed in 200 μl cell lysis buffer (Beyotime) containing 1% PMSF. The cell lysate was centrifuged, and the supernatant was collected. Samples were fractionated by SDS-PAGE electrophoresis and then transferred to PVDF membrane, blocked with 5% non-fat dry milk at room temperature for 5–6 h. The membranes were incubated overnight at 4 °C with primary antibodies diluted in blocking buffer. The membranes were washed three times with TBS-Tween and incubated for 1 h at 37 °C with the respective secondary antibodies diluted in blocking buffer. The membranes were then washed three times with TBS-Tween, and bound proteins were detected using an ECL chromogenic kit (Beyotime).

## Results

### DHAV-1 mediates cell cycle arrest in the S phase

**Fig. 1:**
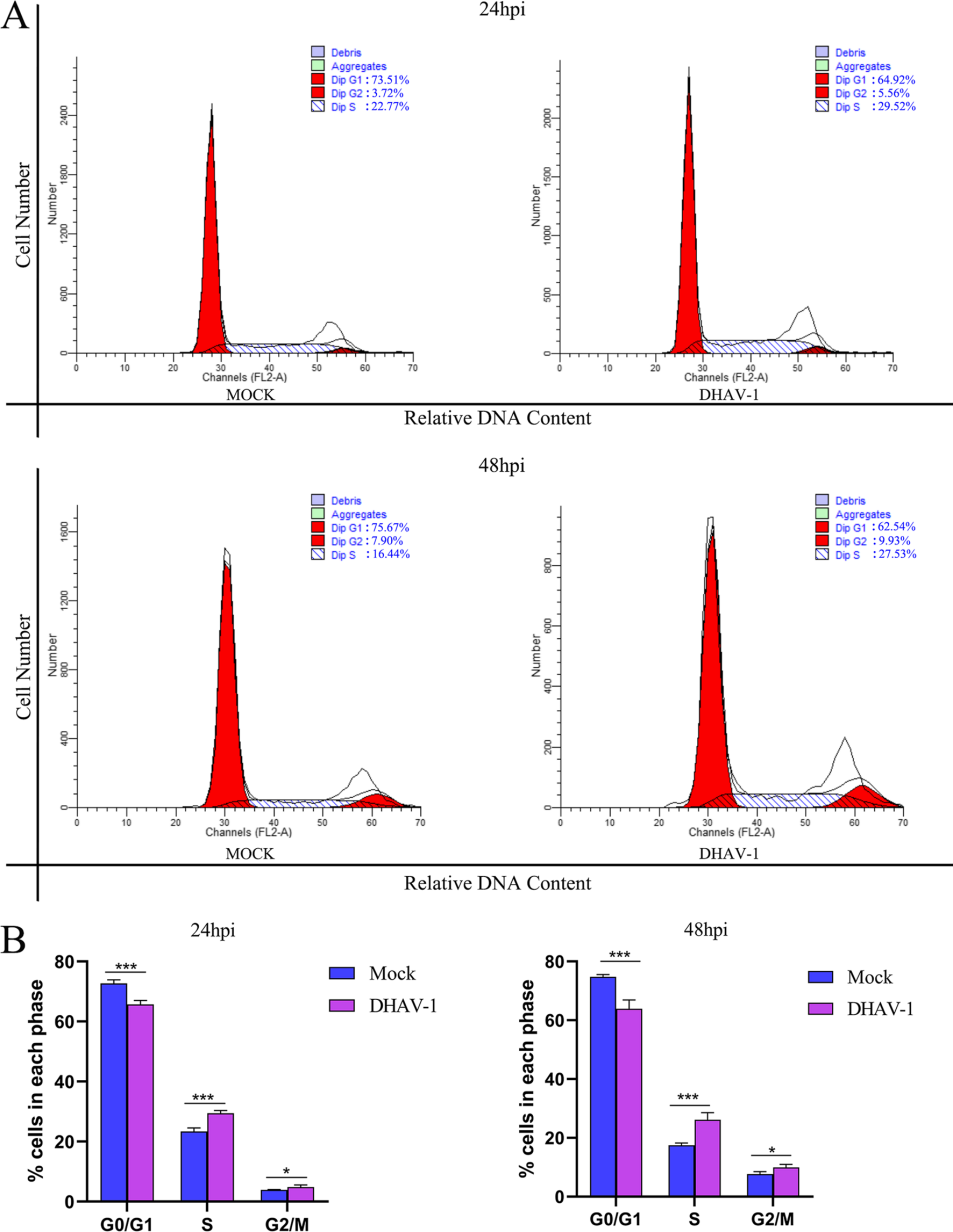
DHAV-1 mediates cell cycle arrest in the S phase. **A** At 24 h or 48hpi, DEFs infected with mock or DHAV-1 at MOI of 1 were collected to analyze cell-cycle profiles by flow cytometry. **B** The histograms were analyzed by the ModFit LT program to display the cell cycle distribution. Differences between the 2 groups were analyzed using Student's *t*-test and were considered significant: *P < 0.05; ***P < 0.001

**Fig. 2:**
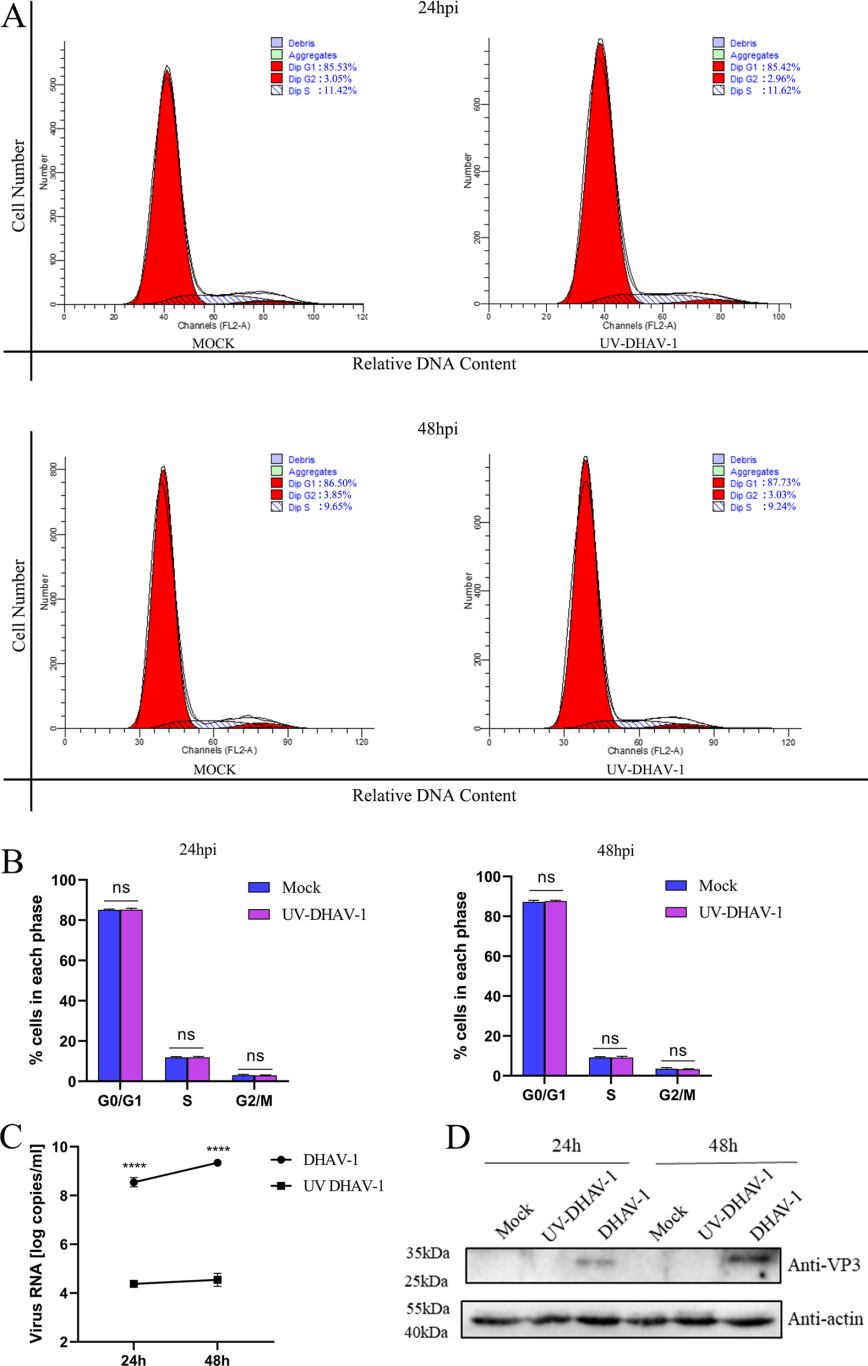
Viral activity is the cause of cell cycle arrest in the S phase. **A** At 24 h or 48hpi, DEFs infected with mock or UV-inactivated DHAV-1 at MOI of 1 were collected to analyze cell-cycle profiles by flow cytometry. **B** The histograms were analyzed by the ModFit LT program to display the cell cycle distribution. **C** DEFs were infected with DHAV-1 and UV-DHAV-1 at MOI of 1, respectively. The X-axis shows the different time points, and the Y-axis represents the logarithm of the number of viral RNA copies. **D** The expression of VP3 protein in DEFs at 24 h or 48hpi. Differences between the 2 groups were analyzed using Student's *t*-test and considered significant at ****P < 0.0001

The cell cycle of DEFs was analyzed by flow cytometry at 24 or 48 h post-infection (hpi) to detect whether DHAV-1 regulates the cell cycle of DEFs (Fig. 1A). Through ModFit analysis (Fig. 1B), at 24 hpi, DEFs in the S phase increased from 23.25 ± 1.21% to 29.26 ± 1.07% (increased by 25.85%, *P* < 0.001); 48 hpi, DEFs in the S phase increased from 17.34 ± 0.9% to 26.74 ± 2.02% (increased by 54.21%, *P* < 0.001). These data indicate that DHAV-1 infection causes the DEFs cell cycle arrest in the S phase. Meanwhile, we used UV to inactivate DHAV-1 and then infected DEFs at MOI of 1. As shown in Fig. 2C, D, compared to the DHAV-1 group, the UV-inactivated DHAV-1 had no increase in virus copy number, and no VP3 expression was detected, indicating that the UV-inactivated DHAV-1 lost the ability to replicate. Compared with the mock group, UV-inactivated DHAV-1 infection could not induce the cell cycle arrest of DEFs in the S phase (Fig. 2A, B).

### S phase arrest promotes the replication of DHAV-1

**Fig. 3:**
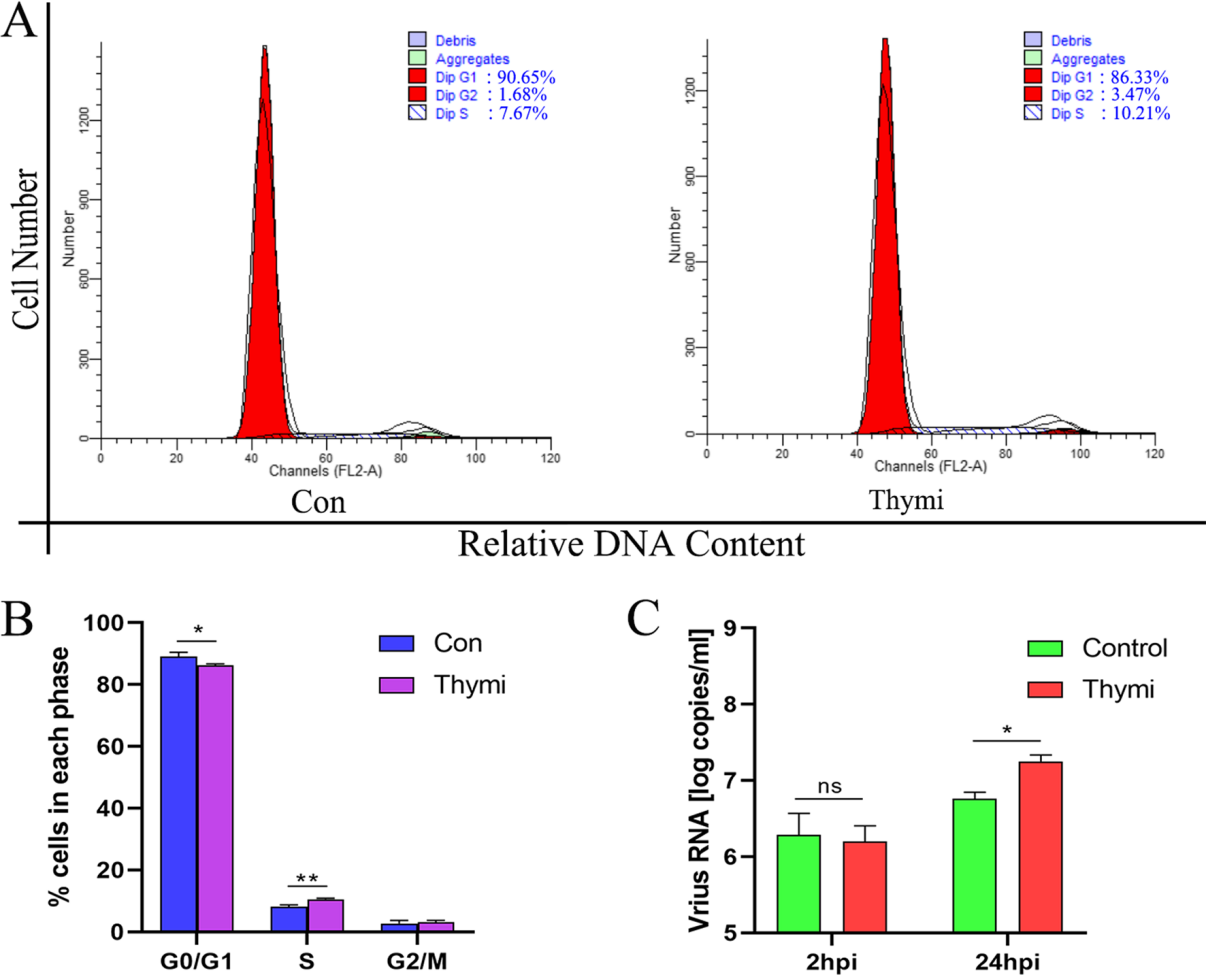
S phase arrest promotes the replication of DHAV-1. **A** After 24 h of Thymine treatment of DEFs, the cell cycle profile was analyzed by flow cytometry. **B** The histograms were analyzed by the ModFit LT program to display the cell cycle distribution. **C** The viral copies were detected at 2 and 24 hpi in the S phase-synchronized or non-synchronized cells. Differences between 2 groups were analyzed using Student's *t*-test and considered significant: *P < 0.05; **P < 0.01

Since DHAV-1 infection causes the DEFs cell cycle arrest in the S phase, we wonder whether the S phase is beneficial to virus replication. The DEFs were treated with 1.0 mM Thymidine for 24 h to make more DEFs synchronization in the S phase, and the cell cycle distribution was detected by flow cytometry. As shown in Fig. 3A, B, compared with the control group, Thymidine treatment increased DEFs in the S phase from 8.24 ± 0.57% to 10.505 ± 0.295% (increased by 27.49%, *P* < 0.01). After treating DEFs with Thymidine for 24 h, the cells were infected with MOI of 0.1 DHAV-1, and viral copy numbers were detected at 2 and 24 hpi. As shown in Fig. 3C, there was no difference in viral copy number after 2 h of infection, while the viral copy number of the Thymidine-treated group was higher than that of the control group at 24 hpi. These results indicate that S phase arrest will not affect the entry of DHAV-1 into cells but will promote the replication of DHAV-1.

### G0/G1 phase arrest also promotes the replication of DHAV-1

**Fig. 4:**
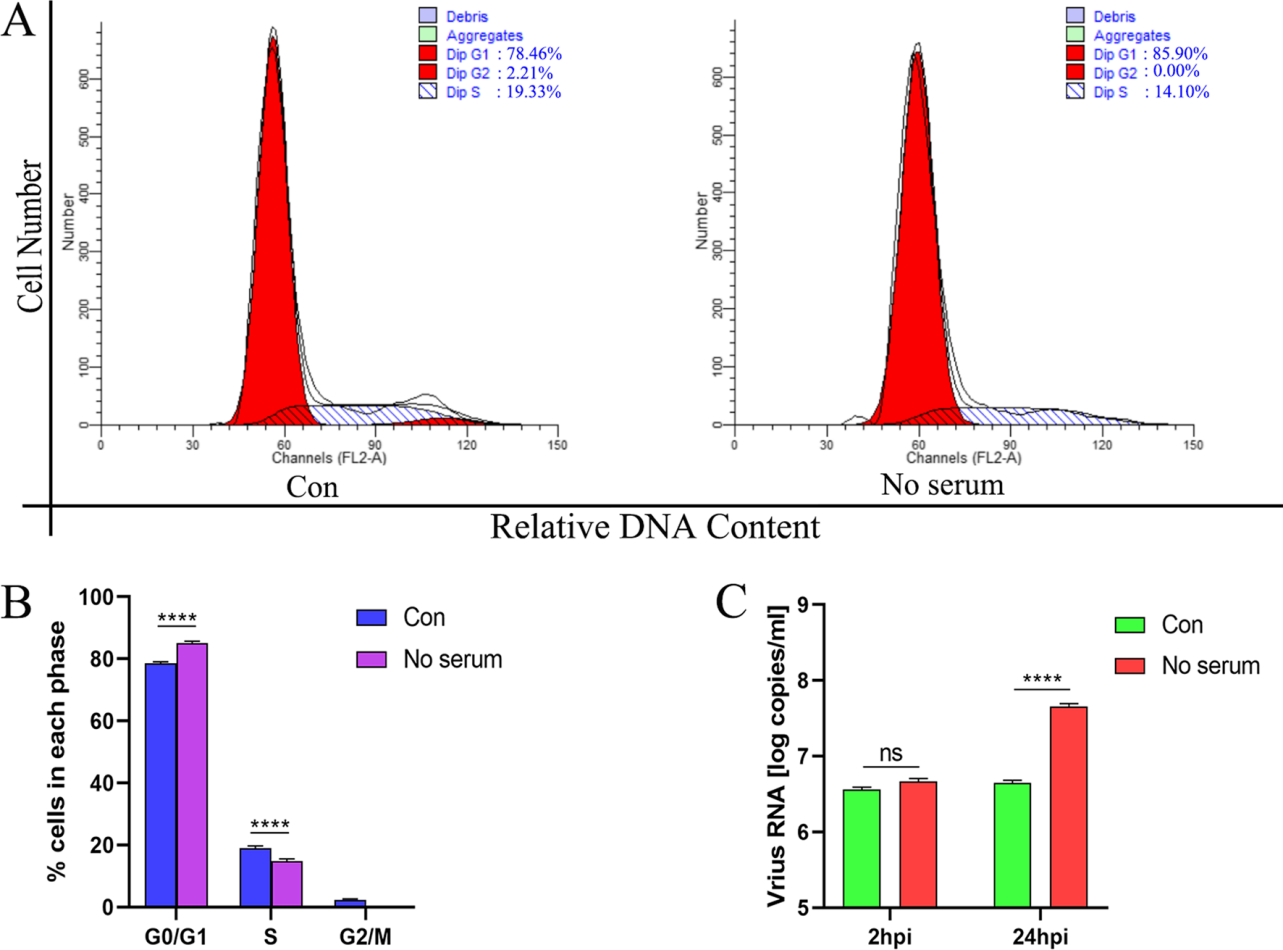
G0/G1 phase arrest also promotes the replication of DHAV-1. **A** DEFs were cultured in a serum-free medium for 24 h, and the cell cycle profile was analyzed by flow cytometry. **B** The histograms were analyzed by the ModFit LT program to display the cell cycle distribution. **C** The viral copies were detected at 2 and 24 hpi in G0/G1 phase-synchronized or non-synchronized cells. Differences between 2 groups were analyzed using Student's *t*-test and were considered significant: ****P < 0.0001

The DEFs were treated with a serum-free medium for 24 h to make more DEFs synchronization in the G0/G1 phase, and the cell cycle distribution was detected by flow cytometry. As shown in Fig. 4A, B, compared with the control group, the treatment of serum-free medium increased DEFs in the G0/G1 phase from 78.645 ± 0.585% to 85.26 ± 0.64% (increased by 8.41%, *P* < 0.0001). After treating DEF cells with a serum-free culture medium for 24 h, the cells were infected with MOI of 0.1 DHAV-1, and viral copy numbers were detected at 2 and 24 hpi. As shown in Fig. 4C, there was no difference in viral copy number after 2 h of infection, while the viral copy number of the serum-free medium treatment group was significantly higher than that of the control group 24 hpi. These results indicate that G0/G1 phase arrest will not affect the entry of DHAV-1 into cells but will promote the replication of DHAV-1.

### G2/M phase arrest does not affect the replication of DHAV-1

**Fig. 5:**
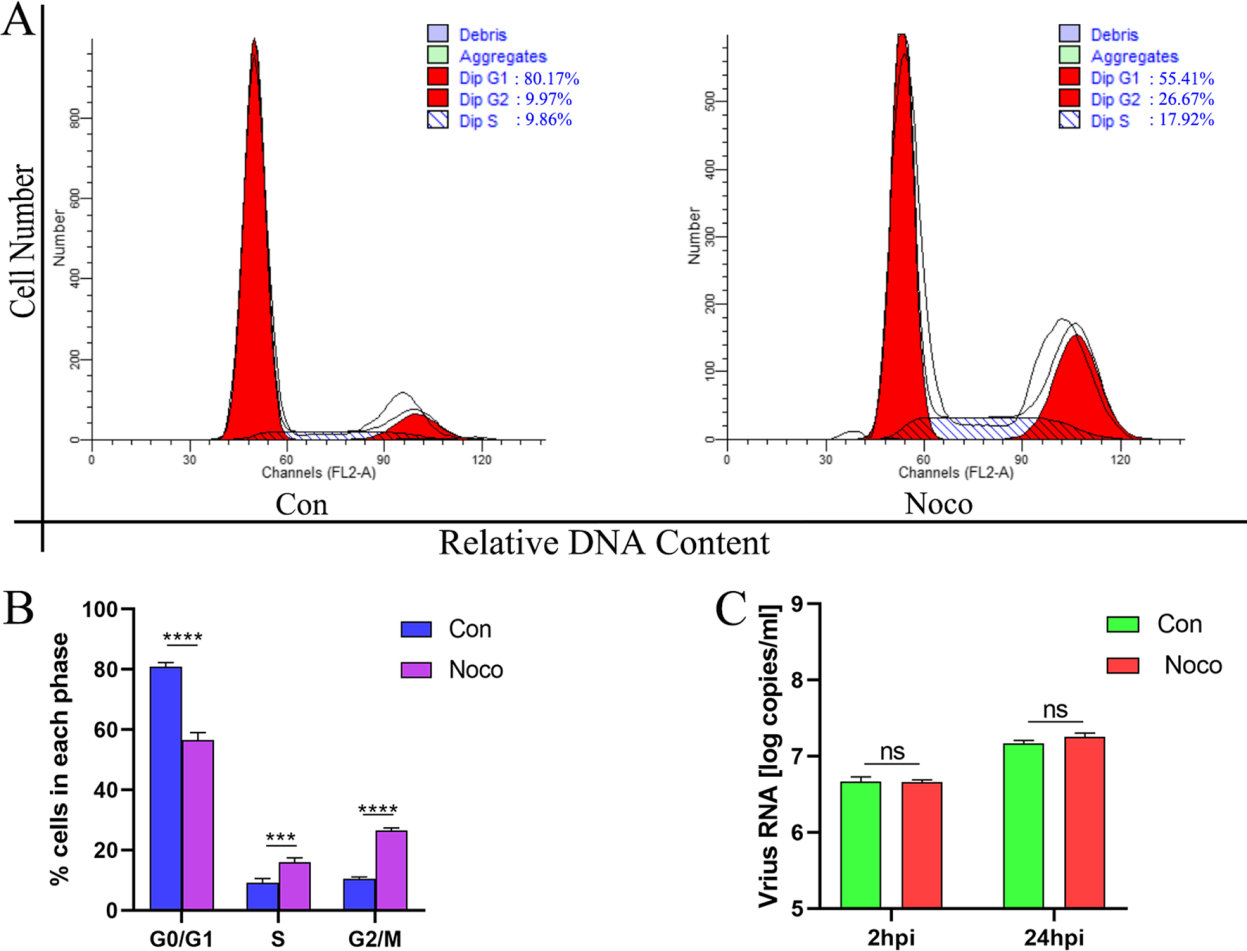
G2/M phase arrest does not affect the replication of DHAV-1. **A** After 24 h of Nocodazole treatment of DEFs, the cell cycle profile was analyzed by flow cytometry. **B** The histograms were analyzed by the ModFit LT program to display the cell cycle distribution. **C** The viral copies were detected at 2 and 24 hpi in G2/M phase-synchronized or non-synchronized cells. Differences between 2 groups were analyzed using Student's *t*-test and were considered significant: ***P < 0.001; ****P < 0.0001

DEFs were treated with 25 ng/ml Nocodazole for 24 h to make more DEFs synchronization in the G2/M phase, and the cell cycle distribution was measured by flow cytometry. As shown in Fig. 5A, B, compared with the control group, Nocodazole treatment increased DEFs in the G2/M phase from 10.52 ± 0.55% to 26.465 ± 0.855% (increased by 151.57%, *P* < 0.0001). After treating DEFs with Nocodazole for 24 h, the cells were infected with MOI of 0.1 DHAV-1, and viral copy numbers were detected at 2 and 24 hpi. As shown in Fig. 5C, there was no difference in viral copy number between the Nocodazole treatment group and the control group after 2 and 24 h infection. These results indicate that G2/M phase arrest does not affect the entry of DHAV-1 into cells, nor does it affect the replication of DHAV-1.

### 3D protein causes the DEFs cell cycle arrest in the S phase

**Fig. 6:**
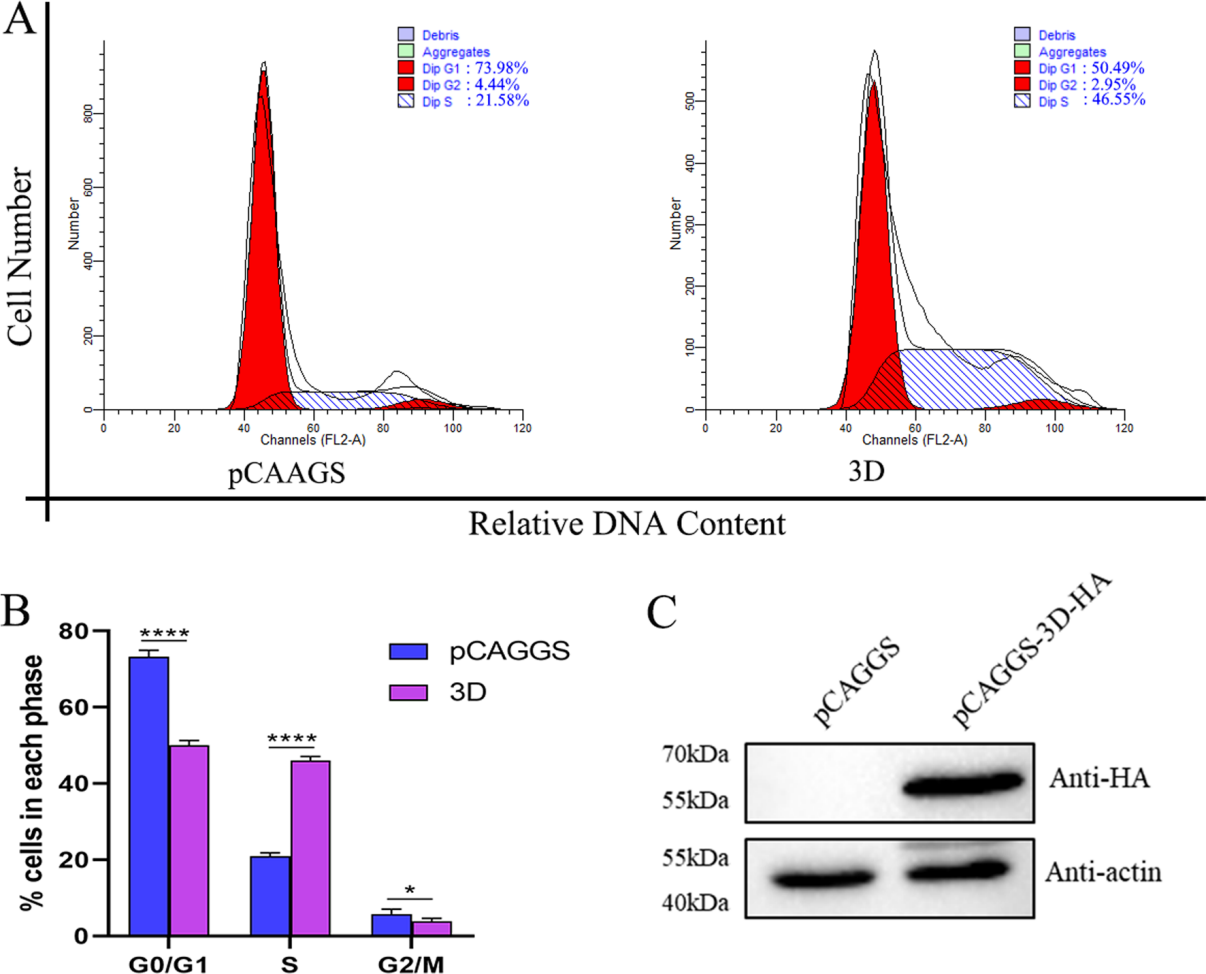
3D protein causes the DEFs cell cycle arrest in the S phase. **A** After 36 h of transfection, DEFs were collected for analyzing cell-cycle profiles by flow cytometry. **B** The histograms were analyzed by the ModFit LT program to display the cell cycle distribution. **C**The expression of the 3D protein in DEFs. Differences between 2 groups were analyzed using Student's *t*-test and were considered significant: *P < 0.05; ****P < 0.0001

In other picornaviruses, cell cycle changes are related to the 3D protein of the virus [11, 12]. However, it is still unknown whether the non-structural protein 3D of DHAV-1 also has such a function. DEFs were transfected with a plasmid expressing the 3D protein and set pCAGGS as a control simultaneously (Fig. 6C). After 36 h of transfection, the cell cycle was detected by flow cytometry (Fig. 6A). Through ModFit analysis (Fig. 6B), compared to the control group, 3D protein increased DEFs in the S phase from 20.86 ± 0.92% to 46.5 ± 0.75% (increased by 122.91%, *P* < 0.0001). These results indicate that the viral non-structural proteins 3D causes the DEFs cell cycle arrest in the S phase.

## Discussion

Regulating the cell cycle to create a favorable environment for virus replication is one of the strategies commonly used by most viruses. However, DHAV-1, as an important pathogen that harms the duck industry, has not been thoroughly studied in the cell cycle regulation. This study showed that DHAV-1 induces DEFs cell cycle arrest in the S phase (Figs. 1, 2). The S phase is the DNA synthesis phase in the cell cycle and requires the participation of various enzymes in the cell. DHAV-1 blocks DEFs in this phase and creates an environment for its replication. In addition, this study also indicates that 3D protein is the cause of cell cycle arrest in the S phase (Fig. 6), which is consistent with the results of other picornaviruses, and may be related to the 3D protein uridylation [11, 12].

Previous reports have demonstrated that DHAV-1 can induce increased phosphorylation of eIF2α [28]. eIF2α phosphorylation plays an important role in viral infection, related to the G0/G1 phase [31]. Newcastle disease virus (NDV) and Muscovy duck reovirus (MDRV) can arrest the G0/G1 phase of cells through the PERK-eIF2α pathway [25, 32]. However, our results show that DHAV-1 does not block the DEFs cell cycle in the G0/G1 phase. The similar result was also reported in EV71 [11, 33].

Since DHAV-1 caused cells to accumulate in the S phase, the cells in the G0/G1 phase in the infection group were significantly lower than the mock group, while the cells in the G2/M phase were slightly higher than the mock group (Fig. 1B). Similarly, the 3D protein causes cells to accumulate in the S phase, but the cells in the G2/M phase are slightly lower than the control group (Fig. 6B), suggesting that viral infection is more complicated than the expression of a single protein and further research is needed to clarify this issue. The situation is similar to that of the Duck Tembusu virus (DTMUV), a single-stranded positive-stranded RNA virus [34].

In picornaviruses, different viruses manipulate the cell cycle differently. CVA6 inhibits cells from G0/G1 phase to S phase [12], while EV71 and CVA16 prevent the cell cycle from transitioning from the S phase to the G2/M phase [11]. These results indicate that G0/G1 phase or S phase arrest is a common strategy used by picornaviruses. In this study, both the S phase and G0/G1 phase are beneficial to DHAV-1 replication. This result is inconsistent with other picornaviruses that specifically block a certain cell cycle stage, implying that after DHAV-1 infection, although the number of cells in the S phase increased by 54.21%, most of the DEFs were still in the G0/G1 phase. This part of the cells in the G0/G1 phase may also be indispensable for DHAV-1 replication because the virus replicates more significantly in the cells synchronized with the G0/G1 phase than the cells synchronized with the S phase (Figs. 3C, 4C). Meanwhile, considering that the rapid multiplication of DHAV-1 may affect the results of drug treatment and cause no significant difference between the experimental group and the control group. Therefore, when exploring the effect of each cycle (S, G0/G1, G2/M) on virus multiplication, we used MOI of 0.1 DHAV-1 to infect DEFs instead of MOI of 1. In addition, viral infection and viral protein overexpression may have different results on the cell cycle. For example, ZIKV infection leads to cell cycle arrest in the S phase [13], while overexpression of its E protein induces G2/M phase arrest [24]. Although the results of this study indicate that both the expression of 3D protein and DHAV-1 infection affect the progress of the S phase, whether other viral proteins affect the G0/G1 phase needs further proof.

## Conclusion

The current study innovatively found the DHAV-1 infection caused the DEFs cell cycle arrest in the S phase. Furthermore, the synchronization of the S phase and the G0/G1 phase is conducive to the replication of DHAV-1, and 3D protein is one of the reasons for cell cycle arrest in the S phase. These results provide basic data for further research on the pathogenic mechanism of DHAV-1.

## Data Availability

The datasets used and analyzed during the current study are available from the corresponding author on reasonable request.

## Abbreviations

DHAV-1: Duck hepatitis A virus type 1
DEFs: Duck Embryo Fibroblasts
Real-time RT-qPCR: Real-time reverse transcriptase quantitative polymerase chain reaction
CDK: Cyclin-dependent kinase
5′ UTR: 5′ Untranslated region
ORF: Open reading frame
3′ UTR: 3′ Untranslated region
MEM: Minimum essential medium
PBS: Phosphate-buffered saline
SDS-PAGE: Sodium dodecyl sulfate–polyacrylamide gel electrophoresis
MOI: Multiplicity of infection
B19V: Human parvovirus B19
MVC: Bocavirus minute virus of canines
ZIKV: Zika virus
CVA6: Coxsackievirus A6
CVA16: Coxsackievirus A16
EV71: Enterovirus 71
NDV: Newcastle disease virus
MDRV: Muscovy duck reovirus
DTMUV: Duck Tembusu virus
